# Does the CHA_2_DS_2_-Vasc score predict procedural and short-term outcomes in patients undergoing transcatheter aortic valve implantation?

**DOI:** 10.1136/openhrt-2014-000170

**Published:** 2015-10-22

**Authors:** Tahir Hamid, Tawfiq R Choudhury, Simon G Anderson, Izhar Hashmi, Saqib Chowdhary, David Hesketh Roberts, Douglas G Fraser, Ragheb Hasan, Vaikom S Mahadevan, Richard Levy

**Affiliations:** 1 Manchester Royal Infirmary, Central Manchester University NHS Foundation Trust, Manchester, UK; 2 University Hospital of South Manchester NHS Foundation Trust, Wythenshawe, UK; 3 Institute of Cardiovascular Sciences, University of Manchester, Manchester, UK; 4 Blackpool Teaching Hospitals NHS Foundation Trust, Blackpool, UK

## Abstract

**Background:**

Transcatheter aortic valve implantation (TAVI) is associated with periprocedural and postprocedural morbidity and mortality. Currently, there is a paucity of risk stratification models for potential TAVI candidates. We employed the CHA_2_DS_2_-Vasc score to quantify the risk of 30-day mortality and morbidity in patients undergoing TAVI.

**Methods and results:**

A retrospective analysis of registry data for consecutive patients undergoing TAVI at 3 tertiary centres in Northwest England between 2008 and 2013. The CHA_2_DS_2_-Vasc score and its modification—the R_2_CHA_2_DS_2_-Vasc score, which includes pre-existing renal impairment and pre-existing conduction abnormality (right bundle branch block/left bundle branch block, RBBB/LBBB)—were calculated for all patients. A total of 313 patients with a mean age of 80 (79.1–80.8) years underwent TAVI. The implanted devices were either the CoreValve or the Edwards-SAPIEN prosthesis. The 30-day mortality was 14.3% in those with a CHA_2_DS_2_-Vasc score ≥6, whereas it was only 6.2% in those with a score <6 (p=0.04). Using the R_2_-CHA_2_DS_2_-Vasc score, the difference was more pronounced with a 30-day mortality of 22.6% in those patients with an R_2_-CHA_2_DS_2_-Vasc score ≥7 compared to 6.0% in those with a R_2_-CHA_2_DS_2_-Vasc score <7 (p=0.001). In multivariable Cox regression analyses, there was a significant and independent relationship between the CHA_2_DS_2_-Vasc score (hazard ratio (HR)= 2.71, (1.01 to 7.31); p<0.05) and the modified R_2_CHA_2_DS_2_-Vasc score (HR=4.27 (1.51 to 12.07); p=0.006) with 30-day mortality.

**Conclusions:**

Our study demonstrates the potential use of the CHA_2_DS_2_-Vasc or the R_2_CHA_2_DS_2_-Vasc score to quantify the risk of mortality in patients undergoing TAVI. This could have significant implications in terms of clinical as well as patients’ decision-making.

**Key messages**

**What is already known about this subject?**

- Severe symptomatic aortic stenosis carries a dismal prognosis without aortic valve replacement. Transcatheter aortic valve implantation (TAVI) has increasingly established itself as a treatment modality for patients unsuitable for surgical valve replacement. However, the risk of mortality and complications post-TAVI is still considerable. There is a paucity of risk stratification scores in potential candidates for TAVI to predict mortality and morbidity post-TAVI.

**What does this study add?**

- This study finds an association between the well-known CHA_2_DS_2_-Vasc score and the risk of 1-year mortality post-TAVI. It also finds an even stronger association between a modified CHA_2_DS_2_-Vasc score (R_2_CHA_2_DS_2_-Vasc) and the risk of 1-year mortality. Thus, this study adds two new risk stratification tools that may be useful in TAVI candidates. The advantage of using the CHA_2_DS_2_-Vasc score is that it is already very well established, easy to use and very well known globally.

**How might this impact on clinical practice?**

- The use of the CHA_2_DS_2_-Vasc score has significant implications in terms of clinical decision-making as to whether a patient is suitable for TAVI, and also for patients’ decision-making.

## Introduction

Severe symptomatic aortic stenosis carries a dismal prognosis without aortic valve replacement, with a mortality rate of around 25% at 1 year.^1^ Surgical aortic valve replacement has been the gold standard of treatment in these patients. However, there are patients within this group with multiple comorbidities who are usually deemed unfit for surgery. In the past, this latter group was managed conservatively^2^ or with aortic valvuloplasty as a palliative measure.^3^ ^4^ Since Cribier performed the first human case of percutaneous aortic valve replacement, transcatheter aortic valve implantation (TAVI) has been an option for patients declined valve replacement surgery. However, TAVI is associated with periprocedural and postprocedural morbidity and mortality.^5–7^ In the PARTNER trial,^8^ ^9^ a prospective, randomised trial, although TAVI was non-inferior to surgical aortic valve replacement in high-risk patients and superior to medical management in non-surgical candidates, the mortality rate at 1-year post-TAVI was still 24.2% and 30.7%, respectively. At present, there is a lack of an established TAVI-specific scoring system to stratify patients who are potentially at high periprocedural risk. Currently, scores such as the European System for Cardiac Operative Risk Evaluation (EUROSCORE), EUROSCORE II and Society of Thoracic Surgeons (STS) are used to risk stratify these high-risk cohorts of patients undergoing surgical aortic valve replacement.^10–12^ In our study, we have used the CHA_2_DS_2_-Vasc score^13^ and a modified version—the R_2_-CHA_2_DS_2_-Vasc score—as potential prognostic tools to quantify the risk of 30-day mortality in patients undergoing TAVI. The CHA_2_DS_2_-Vasc score is a well-validated score to establish the risk of cerebrovascular accident (CVA) in patients with atrial fibrillation.^13^

## Materials and methods

This is a retrospective analysis of prospective data registry of consecutive patients who underwent TAVI at three tertiary centres (Manchester Royal Infirmary, Blackpool Victoria Hospital and University Hospital of South Manchester). Patients received either the CoreValve (Medtronic, Minneapolis, Minnesota, USA) or the Edwards-SAPIEN (Edward Lifesciences, Irvine, California, USA) prosthesis. We obtained data for patients admitted to any of the three sites for implantation of a TAVI between April 2008 and April 2013. Information was collected through correspondence from outpatient clinic visits and the hospitals’ TAVI database, retrospectively.

### Primary end point mortality

The primary clinical end point was all-cause mortality (as recommended by the Valve Academic Research Consortium-VARC,^14^) within 30 days after hospital admission. National unique identifiers were used to link patients with the Office for National Statistics in the UK. We accessed these registries to ascertain the date of death.

### Secondary end points

The secondary end points were mortality within 1 year, stroke, need for a permanent pacemaker insertion within 30 days of the TAVI, major vascular complications (defined as requiring a vascular surgical input, excessive procedure-related bleeding (defined as life-threatening, disabling or major bleeding, fatal bleeding or need for blood transfusion)) and requirement for surgical intervention peri-TAVI or post-TAVI.

### CHA_2_DS_2_-Vasc scoring

We calculated the CHA_2_DS_2_-Vasc score (table 1) for all patients. A modified score (R_2_CHA_2_DS_2_-Vasc), devised by our group, was derived where R_2_ defines pre-existing renal impairment (serum creatinine >200 µmol/L) and pre-existing conduction abnormality (right bundle branch block/left bundle branch block, RBBB/LBBB) on preprocedural ECGs, with one point given for each factor. Patients with a pre-existing permanent pacemaker scored 0 for the rhythm score. The scores were then analysed for any association with 30-day all-cause mortality and the secondary end points.

**Table 1:** CHA_2_DS_2_-Vasc and R_2_-CHA_2_DS_2_-Vasc scoring

	Condition	Points
C	Congestive heart failure	1
H	Hypertension	1
A_2_	Age ≥75 years	2
D	Diabetes	1
S_2_	Prior stroke or TIA	2
V	Vascular disease (eg, peripheral artery disease, myocardial infarction	1
A	Age 65–74 years	1
Sc	Sex category (ie, female gender)	1
R	Renal impairment	1
R	RBBB/LBBB on 12 lead ECG	1

LBBB, left bundle branch block; RBBB, right bundle branch block; TIA, transient ischaemic attack.

### Statistical analysis

The data were analysed with the statistical package Stata/MP V.13.1 (Stata Corp). Anthropometric data are expressed as arithmetic mean (95% CIs). The normal distribution of the continuous variables was examined with Shapiro-Wilk's test. Comparisons of means or proportions were made by the t test on log-transformed data, analysis of variance (ANOVA) or χ^2^ test where relevant. Receiver operating characteristic analysis and the area under the curve were used to identify the sensitivity and specificity of the CHA_2_DS_2_-Vasc and R_2_CHA_2_DS_2_-Vasc cut-off points for mortality. The optimal cut-off values were defined by Youden's index, that is the point at which the value of ‘sensitivity + specificity – 1’ was maximal (CHA_2_DS_2_-Vasc=6 and R_2_CHA_2_DS_2_-VASC=7). The Cox proportional hazard regression models were used to assess the association between the dependent variables and mortality. Multivariable models were adjusted. We checked the proportional hazards assumption for each model based on the analysis of Schoenfield residuals. We compared the predictive powers of the survival models using Harrell's C statistic.^15^

## Results

The study population was drawn from three hospitals in the northwest of England. A total of 313 patients with severe aortic stenosis underwent TAVI. The mean age of the patient population was 80 (95% CI 79.2 to 80.8) years with 140 (44.7%) patients being women. The women were slightly older and had lower proportions with previous myocardial infarction (MI), coronary artery bypass grafting (CABG), hypertension and peripheral vascular disease (PVD) (table 2); 219 (70%) patients received the CoreValve (Medtronic Inc), while 94 (30%) received the Edwards-SAPIEN (Edward Lifesciences) prosthesis and 44 (14.1%) required valvuloplasty prior to TAVI. Eighty patients (26.7% women) had atrial fibrillation.

**Table 2:** Demographic and periprocedural risk factors

Variable	Male	Female	Total
	n=173 (55.3)	n=140 (44.7)	313
Age (years), mean (95% CI)	80.5 (77.7 to 80.0)	81.4 (80.1 to 82.6)**	80 (79.2 to 80.8)
Diabetes (yes), n (%)	55 (31.8)	31 (22.1)	86 (27.5)
Smoking status, n (%)			
Never-smoker	66 (38.1)	78 (55.7)*	144 (46.0)
Ex-smoker	101 (63.8)	58 (41.4)	159 (50.8)
Current smoker	2 (1.7)	3 (2.1)	6 (1.9)
Unknown	3 (1.7)	1 (0.71)	4 (1.3)
Renal impairment, n (%)	38 (22.0)	19 (13.6)	57 (18.2)
Previous MI, n (%)	55 (31.8)	23 (16.4)*	78 (24.9)
Previous PCI, n (%)	29 (16.8)	24 (17.4)	53 (16.9)
Hypertension, n (%)	133 (76.9)	92 (65.7)*	225 (71.9)
Congestive heart failure, n (%)	62 (35.8)	34 (24.3)*	96 (30.7)
Pulmonary hypertension, n (%)	20 (11.9)	21 (15.8)	41 (13.6)
Previous CABG, n (%)	81 (46.8)	23 (16.4)***	104 (33.2)
Peripheral vascular disease, n (%)	112 (64.7.0)	69 (49.3)**	181 (57.8)
Calcification of ascending aorta, n (%)	41 (23.8)	29 (20.7)	70 (22.4)
Atrial fibrillation, n (%)	43 (24.9)	37 (26.4)	80 (25.6)
Previous valvuloplasty, n (%)	28 (16.2)	16 (11.5)	44 (14.1)
Log EuroScore, mean (95% CI)	21.7 (19.3 to 24.0)	22.0 (19.2 to 24.8)	21.8 (20.0 to 23.6)

*p<0.05; **p<0.01 and ***p<0.001 for differences between proportions or means.

CABG, coronary artery bypass grafting; MI, myocardial infarction; PCI, percutaneous coronary intervention.

Periprocedural complications included 13 (4.2%) patients who had a CVA; 50 (17.5%) patients (of the 285 without pre-existing pacemakers) required permanent pacemakers within 30 days post-TAVI, while 12 (3.8%) had major vascular complications requiring additional interventions, and one had cardiac tamponade (0.3%). Of the 313 individuals at baseline, a total of 96 (30.7%) deaths were recorded during the period of follow-up. Crude mortality rates for both sexes combined were 155.9 (95% CI 127.6 to 190.4) deaths per 1000 person-years.

Preoperative CHA_2_DS_2_-Vasc and R_2_CHA_2_DS_2_-Vasc scores were derived after combining patients from all sites; 56 (17.9%) patients had a CHA_2_DS_2_-Vasc score ≥6 and 31 (9.9%) had a modified R_2_CHA_2_DS_2_-Vasc score ≥7. The distributions by risk factor stratified by the CHA_2_DS_2_-Vasc and R_2_CHA_2_DS_2_-Vasc scores are presented in table 3. There were more women in the higher score category. There was a greater proportion of patients with diabetes, hypertension, congestive heart failure and peripheral vascular disease among those with a CHA_2_DS_2_-Vasc score ≥6 and an R_2_CHA_2_DS_2_-Vasc score ≥7. The log EuroSCORE was also greater in these patients. The proportion of those who smoked or had renal impairment, coronary artery disease and atrial fibrillation was similar.

**Table 3:** Comparison of demographic and periprocedural factors by composite CHA_2_DS_2_-Vasc and the modified R_2_CHA_2_DS_2_-VASC Scores

Variable	CHA_2_DS_2_-Vasc <6	CHA_2_DS_2_-Vasc ≥6	R_2_CHA_2_DS_2_-Vasc <7	R_2_CHA_2_DS_2_-Vasc ≥7
	n=257 (81.1)	n=56 (17.9)	n=282 (90.1)	n=31 (9.9)
Age (years), mean (95% CI)	79.7 (78.2 to 80.6)	81.6 (79.6 to 83.5)	79.7 (78.8 to 80.6)	82.6 (79.9 to 85.2)*
Sex (female), n (%)	103 (40.1)	37 (66.1)***	119 (42.2)	21 (67.7)**
Diabetes (yes), n (%)	58 (22.6)	28 (50.0)***	74 (26.2)	12 (38.7)
Smoking status, n (%)				
Never-smoker	117 (45.5)	27 (48.2)	128 (45.4)	16 (51.6)
Ex-smoker	130 (50.6)	29 (51.8)	144 (51.1)	15 (48.4)
Current smoker	6 (2.3)	–	6 (2.1)	–
Unknown	4 (1.6)	–	4 (1.4)	–
Renal impairment, n (%)	44 (17.1)	13 (23.2)	44 (17.1)	13 (23.2)
Previous MI, n (%)	61 (23.7)	17 (30.4)	71 (25.2)	7 (22.6)
Previous PCI, n (%)	42 (16.3)	11 (19.6)	44 (15.6)	9 (29.0)
Hypertension, n (%)	178 (69.3)	47 (83.9)*	197 (69.9)	28 (90.3)*
Congestive heart failure, n (%)	65 (25.3)	31 (55.4)***	79 (28.0)	17 (54.8)**
Pulmonary hypertension, n (%)	34 (13.8)	7 (12.7)	38 (14.1)	3 (9.7)
Previous CABG, n (%)	82 (31.9)	22 (39.3)	95 (33.7)	9 (29.0)
Peripheral vascular disease, n (%)	135 (52.50	46 (82.1)***	156 (55.3)	25 (80.7)**
Atrial fibrillation, n (%)	63 (24.5)	17 (30.4)	72 (25.5)	8 (25.8)
Calcification of ascending aorta	62 (24.1)	8 (14.3)	64 (22.7)	6 (19.3)
Previous valvuloplasty, n (%)	33 (12.9)	11 (19.6)	39 (13.8)	5 (16.7)
Major vascular complications, n (%)	17 (6.8)	6 (10.7)	19 (6.9)	4 (12.9)
Log EuroScore, mean (95% CI)	19.4 (17.5 to 21.3)	32.7 (28.7 to 36.6)***	20.5 (18.7 to 22.3)	32.9 (27.6 to 38.2)***

*p<0.05; **p<0.01 and ***p<0.001 for differences between proportions or means.

CABG, coronary artery bypass grafting; MI, myocardial infarction; PCI, percutaneous coronary intervention.

### Primary end point: 30-day mortality

There were 24 (7.7%) deaths at 30 days with 8 deaths (14.3%) in patients with a CHA_2_DS_2_-Vasc score ≥6. This was significantly greater compared with patients with a preoperative CHA_2_DS_2_-Vasc score <6 (6.2%; p=0.04). Using the R_2_CHA_2_DS_2_-Vasc score, the 30-day mortality rate was 22.6% in those patients with a R_2_CHA_2_DS_2_-Vasc score ≥7 compared to 6.0% deaths in those with a score <7 (p=0.001).

Patients with a CHA_2_DS_2_-Vasc score ≥6 had twice the likelihood of mortality within 30 days compared with those with a score <6 (HR 2.41 (1.03 to 5.63)). When the R_2_CHA_2_DS_2_-VASC score was considered, the risk of 30-day mortality was four times greater in those with a score ≥7 compared with those with a CHA_2_DS_2_-Vasc score <7 (HR 4.14 (1.71 to 9.98); p<0.01).

### Secondary end points

**Figure 1:**
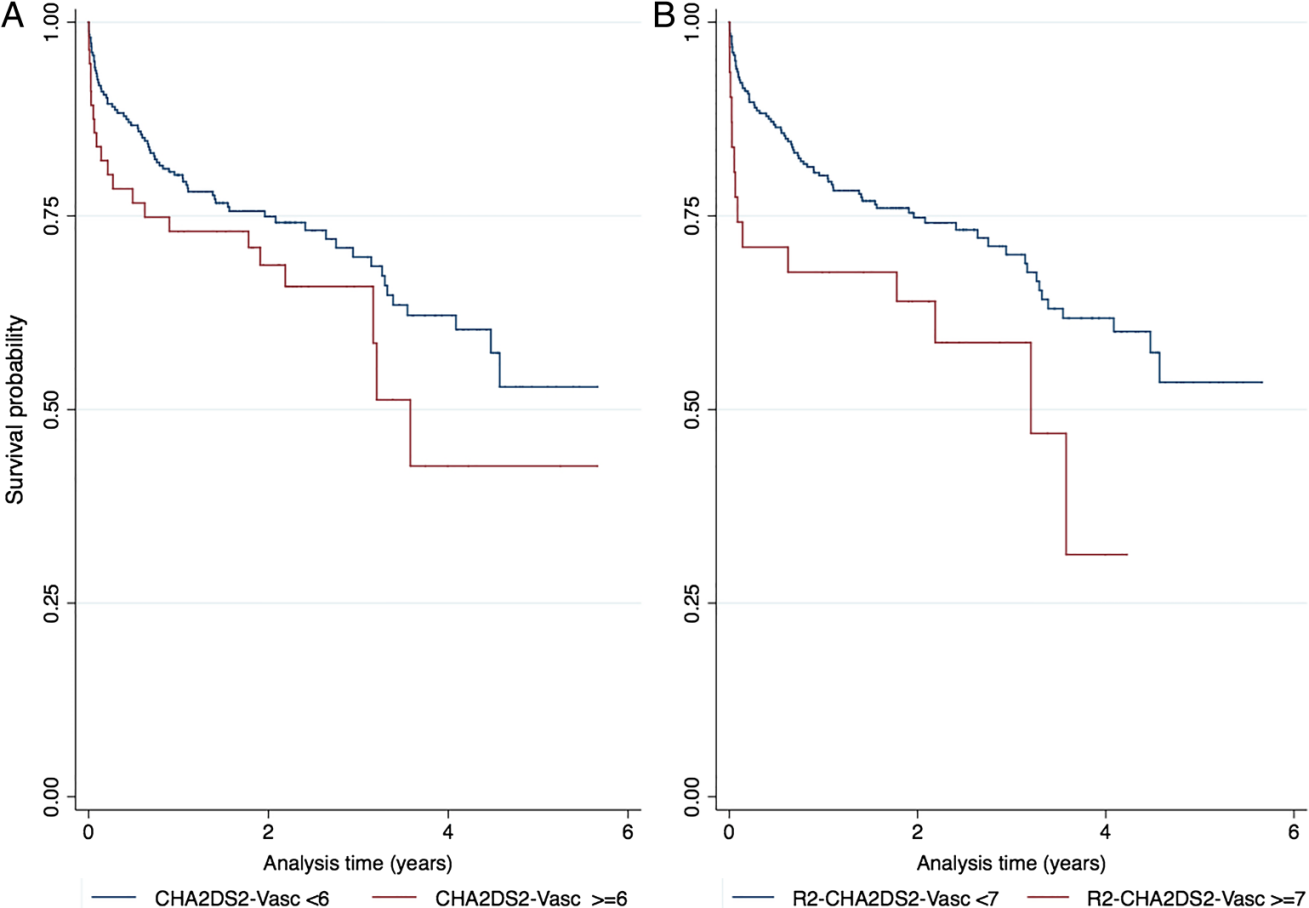
(A) Kaplan–Meier plots of survival curves by CHA_2_DS_2_-Vasc category (<6 vs ≥6) and (B) R_2_CHA_2_DS_2_-Vasc category (<7 vs ≥7).

There were no differences in 1-year mortality proportions for a CHA_2_DS_2_-Vasc score of 6 and <6 (26.8% and 19.5% (p=0.220), respectively). The Kaplan-Meier curves for both groups are shown in figure 1A, B, respectively. Similarly, the proportions of deaths were no different at 1 year using the R_2_CHA_2_DS_2_-Vasc score (33.3% vs 19.5% for a CHA_2_DS_2_-Vasc score ≥7 and <7, respectively (χ^2^=2.76; p=0.097)). In those patients who had a preoperative CHA_2_DS_2_-Vasc score >6, the overall mortality rates were greater than those with a CHA_2_DS_2_-Vasc score <6 (201 (131.0 to 308.3) versus 146.6 (116.9 to 183.8) per 1000 person-years). Table 4 shows a comparison of risk factors by mortality status. In univariable Cox regression analyses (table 5), there were no relationships with age, sex, renal impairment, peripheral vascular disease, hypertension, hyperlipidaemia, diabetes history, smoking status, congestive heart failure, previous MI, previous CABG and previous percutaneous coronary intervention (PCI) with 30-day mortality.

**Table 4:** Comparison of demographic and periprocedural risk factors by mortality status

Variable	Alive	Dead
	n=217 (69.3)	n=96 (30.7)
Age (years), mean (95% CI)	80.5 (79.5 to 81.8)	78.9 (77.4 to 80.5)
Sex (female), n (%)	106 (48.8)	34 (35.4)*
Diabetes, n (%)	56 (25.8)	30 (31.2)
Smoking status, n (%)		
Never smoker	105 (48.4)	39 (40.6)
Ex-smoker	105 (48.4)	54 (56.3)
Current smoker	4 (1.8)	2 (2.1)
Unknown	3 (1.38)	1 (1.0)
Renal impairment, n (%)	38 (17.5)	19 (19.8)
Previous MI, n (%)	48 (22.1)	30 (31.3)
Previous PCI, n (%)	30 (13.8)	23 (24.0)*
Hypertension, n (%)	156 (71.9)	69 (71.9)
Congestive heart failure, n (%)	63 (29.0)	33 (34.4)
Pulmonary hypertension, n (%)	28 (13.7)	13 (13.5)
Previous CABG, n (%)	73 (33.6)	31 (32.3)
Peripheral vascular disease, n (%)	122 (56.2)	59 (61.5)
Atrial fibrillation, n (%)	49 (22.6)	31 (32.3)
Calcification of ascending aorta, n (%)	45 (20.7)	25 (26.0)
Previous valvuloplasty, n (%)	25 (11.6)	19 (19.8)
Log EuroScore, mean (95% CI)	20.6 (18.4 to 22.7)	24.6 (21.4 to 27.8)*

*p<0.05; **p<0.01 and ***p<0.001 for differences between proportions or means.

CABG, coronary artery bypass grafting; MI, myocardial infarction; PCI, percutaneous coronary intervention.

**Table 5:** Univariable HRs for mortality at 30 days for all patients

	HR	p Value
Age (per year)	0.97 (0.95 to 0.99)	0.035
Sex (female)	0.64 (0.42 to 0.98)	0.040
Diabetes (yes)	1.27 (0.82 to 1.95)	0.282
Smoking status	1.24 (0.82=1.63)	0.413
Renal impairment	1.09 (0.66 to 1.80)	0.737
Previous MI	1.37 (0.89 to 2.12)	0.149
Previous PCI	1.53 (0.96 to 2.45)	0.073
Previous CABG	1.02 (0.67 to 1.57)	0.916
Hypertension	0.95 (0.61 to 1.48)	0.821
Congestive heart failure	1.24 (0.81 to 1.89)	0.317
Peripheral vascular disease	1.29 (0.85 to 1.95)	0.225
Calcification of ascending aorta	1.25 (0.79 to 1.98)	0.326
Previous valvuloplasty	1.47 (0.89 to 2.43)	0.129
History of atrial fibrillation	1.48 (0.63 to 3.46)	0.366
Log EuroScore (per unit)	0.99 (0.96 to 1.02)	0.671
CHA_2_DS_2_-Vasc (≥6)	2.41 (1.03 to 5.53)	0.042
R_2_CHA_2_DS_2_-Vasc (≥7)	4.13 (1.71 to 9.98)	0.002

CABG, coronary artery bypass grafting; MI, myocardial infarction; PCI, percutaneous coronary intervention.

**Figure 2:**
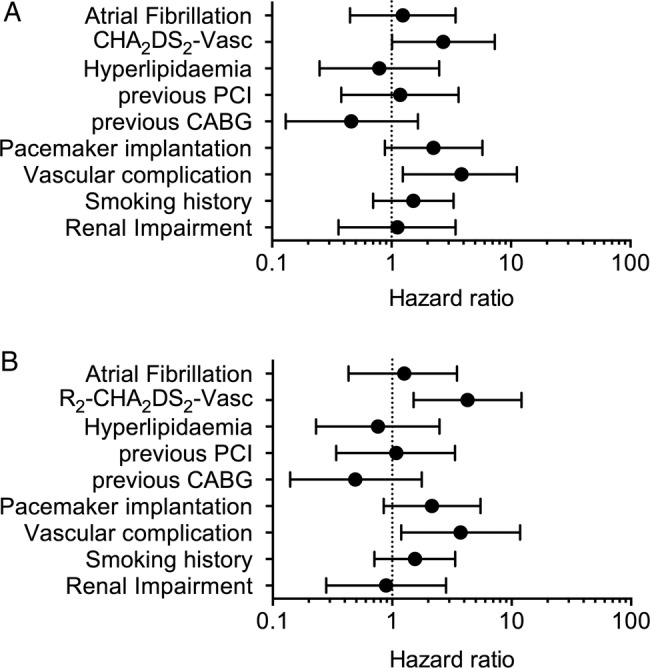
Multivariable Cox regressions showing the independent association of higher (A) CHA_2_DS_2_-Vasc scores (≥6) and (B) R_2_CHA_2_DS_2_-Vasc scores (≥7) with 30-day mortality (CABG, coronary artery bypass grafting; PCI, percutaneous coronary intervention).

In multivariable Cox regression analyses, patients with a CHA_2_DS_2_-Vasc score ≥6 had about three times the risk of death (HR 2.71 (1.01 to 7.31); Harrell's C=0.73) within 30 days. This risk was independent of renal impairment, hyperlipidaemia, smoking history, previous CABG, history of atrial fibrillation, previous PCI periprocedural complications including requiring pacing or vascular complications (figure 2A). In a similar model (figure 2B) replacing the CHA_2_DS_2_-Vasc score with the R_2_CHA_2_DS_2_-Vasc score, this independent relationship remained (HR 4.27 (1.50 to 12.07)), with vascular complications also independently associated with mortality (HR 3.74 (1.19 to 11.77); Harrell’s C=0.75).

**Figure 3:**
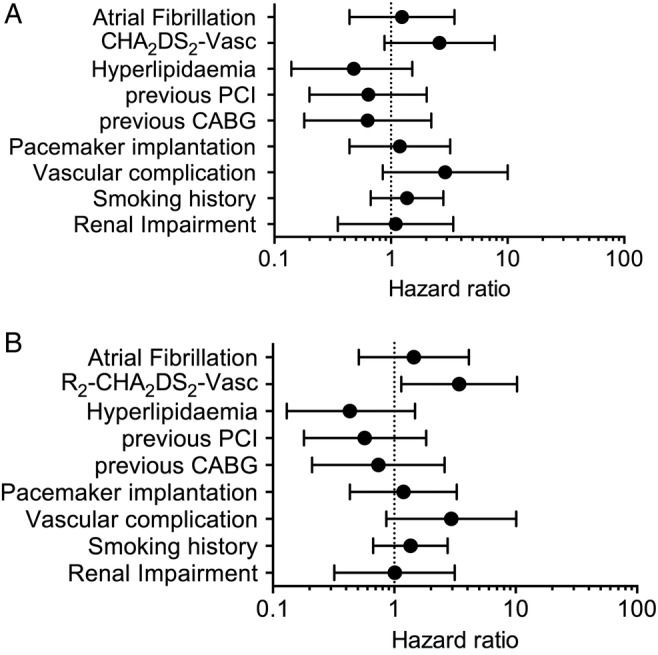
(A) Multivariable Cox regressions showing the association of higher CHA_2_DS_2_-Vasc scores (≥6) and (B) R_2_CHA_2_DS_2_-Vasc scores (≥7) with 1-year mortality (CABG, coronary artery bypass grafting; PCI, percutaneous coronary intervention).

We also performed multivariable Cox regression analyses to quantify the likelihood of death at 1 year. The estimates were attenuated with no increased risk of mortality at 1 year for those with a CHA_2_DS_2_-Vasc score ≥6 compared with those with a score <6 (figure 3A). Patients with an R_2_CHA_2_DS_2_-Vasc score ≥7 had more than three times the increased risk of mortality within a year compared with those with lower scores (figure 3B, Harrell's C=0.79 for model).

## Discussion

TAVI carries significant increased procedural-related mortality and morbidity risks.^10^ ^16–18^ We have shown the potential for employing the well-known CHA_2_DS_2_-Vasc score typically used in predicting stroke in atrial fibrillation, to estimate the likelihood of 30-day all-cause mortality in patients undergoing a TAVI procedure. Furthermore, we have shown that a modified version—the R_2_CHA_2_DS_2_-Vasc score, which adds a point each for renal dysfunction or a history of RBBB/LBBB—is associated with increased mortality at 30 days, and up to 1 year, and may play a role in quantifying the risk of mortality in patients undergoing TAVI.

Currently, the EUROSCORE, EUROSCORE II,^11^ Logistic EuroSCORE^19^ and STS^12^ are used to estimate predicted surgical mortality in cardiac surgery including aortic valve replacement. These scores were originally validated from surgical cohorts in patients who were younger (the patients in the original EUROSCORE cohort had a mean age of 62.5 years) compared with the typically older TAVI patients (mean age 84 and 83 years in the PARTNER TAVI trial cohorts A and B, respectively).^8^ ^9^ The original cohort also included around 40% women, a high proportion of hypertensives (67.3%), 8.5% who had had a CVA/transient ischaemic attack (TIA) and around 17% were with diabetes. These characteristics are very similar to those in our study population (47% women, 57% hypertensive, 12% who had had a stroke or TIA and 18% who were with diabetes).^13^ Although all the EUROSCORE, EUROSCORE II and the Logistic EuroSCORE have shown moderate correlation with short-term mortality after TAVI, they are validated to predict risks from undergoing surgical valve replacement than a percutaneous transcatheter-based procedure such as TAVI.^20^ Second, the STS and EUROSCORE have multiple variables often necessitating online calculators; therefore, the need for a simple non-surgically oriented score is evident.

The CHA_2_DS_2_-Vasc score was originally derived from a medical cohort of patients with non-valvular atrial fibrillation (AF) to predict the risk of thromboembolism.^13^ Recently, this score has also been demonstrated to predict risk of stroke in patients without AF^21^ and coronary artery disease severity,^22^ and has been associated with left atrial dysfunction in patients with coronary heart disease.^23^ Furthermore, the CHA_2_DS_2_-Vasc score includes a number of variables that are relevant to TAVI patients. To account for the presence of renal and conduction abnormalities, we derived a modified version of the CHA_2_DS_2_-Vasc score to take into account baseline renal function and ECG changes of bundle branch block. Renal function at baseline has been shown to be independently associated with mortality post-TAVI.^6^ RBBB pattern on a baseline ECG is associated with the future need for permanent pacemaker implantation post-TAVI.^7^

Our results showed a strong association between the R_2_CHA_2_DS_2_-Vasc score and short-term (30 days) all-cause mortality. Patients with a baseline CHA_2_DS_2_-Vasc score ≥6 or a modified R_2_CHA_2_DS_2_-Vasc score ≥7 appeared to have increased short-term mortality (14.3% vs 22.6%, respectively). While the scores were significantly associated with short-term mortality, only those with higher R_2_CHA_2_DS_2_-Vasc scores had significantly worse prognosis at 1 year. Confirmation of these findings in a larger prospective study or validation of the model in a separate cohort may clarify whether either score has a role in guiding clinical decision-making during the assessment of potential candidates at the TAVI multidisciplinary meetings.

### Study limitations

The findings presented here are novel but are limited by the small sample size and its consequent impact via possible type 2 errors. The current study is a hypothesis-generating retrospective analysis designed to test association between a risk score designed for predicting stroke risk and mortality related to the TAVI procedure. A more comprehensive study would require application of this score to the national UK TAVI database, for example, which would have a greater number of patients. Further studies will be necessary to validate the findings, taking into account other procedural factors. Lastly, we did not undertake a direct comparison between the CHA_2_DS_2_-Vasc score and the established surgical-based risk-scoring tools—EUROSCORE, EUROSCORE II or STS.

## Conclusion

This multicentre retrospective analysis of patients in a TAVI registry demonstrates the potential use of the R_2_CHA_2_DS_2_-Vasc score (and the CHA_2_DS_2_-Vasc score) as a simple tool for the identification of patients at high risk from short-term or medium-term mortality. If validated in a larger prospective cohort, the score may provide both the clinical team and patients with a simple tool to estimate mortality in TAVI procedures.
